# 83. Asymptomatic cardiac arrhythmia in systemic sclerosis

**DOI:** 10.1093/rap/rky034.046

**Published:** 2018-09-20

**Authors:** Fatemah Baron, Rajneet Singh, Bernadette Lynch

**Affiliations:** Rheumatology, Galway University Hospital, Galway, IRELAND

**Introduction:** Systemic sclerosis (SSc) is a chronic autoimmune disease characterised by microangiopathy and excessive cutaneous and visceral fibrosis. Internationally, systemic sclerosis is estimated to occur in 3 to 10 people per one million. Two main variants which are widely recognised are limited cutaneous systemic sclerosis (lcSSc) in which skin sclerosis is limited to distal extremities and diffuse cutaneous systemic sclerosis (dcSSc) which involves sclerosis of skin proximally. Systemic sclerosis can present with protean manifestations and can cause significant organ dysfunction. Cardiac involvement in SSc can virtually effects any structure and when symptomatic, it predicts a poor prognosis. Here, we describe a case of 37 year old male with established diagnosis of diffuse systemic sclerosis who presented with asymptomatic tachyarrhythmia.

**Case description:** 37 year male with 15 pack year history of smoking and a background of hypertension, presented to rheumatology clinic with generalised arthralgia without any other features of CTD or inflammatory arthritis. Examination was unremarkable and laboratory workup revealed mildly positive anti-SCL 70 antibody around 14 (normal <7) by ELISA. On follow-up review, clinical examination was consistent with sclerodactyly, calcinosis and Raynaud’s phenomenon. Repeat antibody screen showed positive antinuclear antibodies (ANA) > 1280, anti- extracted nuclear antibodies (ENA) >34.0 and anti- SCL 70 was significantly raised with level of 240 by ELISA. He was started on hydroxychloroquine 400mg daily and nifidipine 20mg TDS. Over a six month period, the patient developed diffuse skin thickening with modified Rodnan skin score (MRSS) of 21/51. He also described history of intermittent dysphagia, dyspnoea and worsening of Raynaud’s phenomenon associated with digital ulceration. Pulmonary function tests showed restrictive pattern with FVC of 66%, FEV1 of 66% and FEV1/FVC- 82% with reduced DLCO of 56 percent. Barium swallow showed severe oesophageal reflux to mid thorax and dilated oesophagus. Oesophageal gastroscopy (OGD) was consistent with lower oesophageal ulcer and gastritis. His medications were optimised and he was commenced on proton pump inhibitor and azathioprine. Patient was further admitted for ilioprost infusion to treat active digital ulcers and worsening Raynaud’s phenomenon. During routine observations, he was found to have tachycardia, electrocardiogram (ECG) showed atrial flutter at a rate of 150 bpm with troponin T and proBNP measurements of - 137ng/L (0- 14ng/L) and 3019 ng/L (0 - 86 ng/L) respectively. Patient denied any chest pain, palpitations or orthopnoea. Subsequently, he developed supraventricular tachycardia (SVT) at rate of 200 bpm, he was normotensive and asymptomatic. Pharmacological cardioversion failed and patient continued to have intermittent atrial flutters with SVT and underwent two unsuccessful DC cardioversions. Echocardiogram was consistent with severe ventricular impairment, EF of 40%, and extensive posterior, basal and mid inferior wall akinesia with no evidence of valvular heart disease. Estimated PASP was 25mmhg. Coronary angiogram and right heart catheterisationshowed normal coronary structure and no evidence of pulmonary hypertension. Cardiac MRI with gadolinium contrast showed multiple pattern fibrosis with significant bi-ventricular impairment due to myocardial fibrosis and global myocardial oedema with LVEF of 33% and RVEF of 24%. MRI findings were suggestive of cardiac scleroderma. The patient was commenced on anti-arrhythmic, sotalol, and life-long anticoagulation, apixiban. Patient was discharged home in view to have implantable cardioverter defibrillator (ICD) for primary prevention of ventricular arrhythmia and sudden death in setting of severe cardiomyopathy and he is plan further for intravenous cyclophosphamide.

**Discussion:** Cardiac involvement in systemic sclerosis can be manifested as a direct myocardial, pericardial or conduction system abnormalities and majority of these patients may remain subclinical. The estimated clinical prevalence is about 15-35% . Furthermore, sudden cardiac death (SCD) is reported in 5-21% of patients with SSc. The literature specific to cardiac scleroderma and strategies on screening and diagnosing, unlike pulmonary hypertension and interstitial lung disease is sparse. We present this case to contribute to the experience of diagnosing and managing cardiac complications in SSc. To successfully manage cardiac scleroderma, it requires a careful screening, a high index of suspicion and a multidisciplinary approach. Anti-arrhythmic therapy is the mainstay of management with the use of pacemaker and ICD in more severe life threatening arrhythmias. To the best of our knowledge, no randomized controlled trials have compared efficacy of anti-arrhythmic drugs to treat conduction abnormalities in SSc cohort. Thus, medication selection is tailored to the individual patient and the type of arrhythmia.

**Key Learning Points:** Sudden death could happen in up to 20% of SSc patients, therefore it is important to screen SSc population for cardiac involvement. Greater awareness of SSc manifestations and complications should necessitate risk stratification. Further research is required which may allow focused guidance for early diagnosing and monitoring of these patients with cardiac involvement.

**Disclosure: F. Baron:** None.

